# Demographic, clinical characteristics and treatment outcomes of immune-complex membranoproliferative glomerulonephritis and C3 glomerulonephritis in Japan: A retrospective analysis of data from the Japan Renal Biopsy Registry

**DOI:** 10.1371/journal.pone.0257397

**Published:** 2021-09-14

**Authors:** Naoki Nakagawa, Masashi Mizuno, Sawako Kato, Shoichi Maruyama, Hiroshi Sato, Izaya Nakaya, Hitoshi Sugiyama, Shouichi Fujimoto, Kenichiro Miura, Chieko Matsumura, Yoshimitsu Gotoh, Hitoshi Suzuki, Aki Kuroki, Atsunori Yoshino, Shinya Nakatani, Keiju Hiromura, Ryohei Yamamoto, Hitoshi Yokoyama, Ichiei Narita, Yoshitaka Isaka

**Affiliations:** 1 Division of Cardiology, Nephrology, Pulmonology and Neurology, Department of Internal Medicine, Asahikawa Medical University, Asahikawa, Japan; 2 Department of Nephrology, Nagoya University Graduate School of Medicine, Nagoya, Japan; 3 Renal Replacement Therapy, Nagoya University Graduate School of Medicine, Nagoya, Japan; 4 Clinical Pharmacology and Therapeutics, Tohoku University, Graduate School of Pharmaceutical Sciences, Sendai, Japan; 5 Department of Nephrology and Rheumatology, Iwate Prefectural Central Hospital, Morioka, Japan; 6 Department of Human Resource Development of Dialysis Therapy for Kidney Disease, Okayama University Graduate School of Medicine, Dentistry and Pharmaceutical Sciences, Okayama, Japan; 7 Faculty of Medicine, Department of Hemovascular Medicine and Artificial Organs, University of Miyazaki, Miyazaki, Japan; 8 Department of Pediatric Nephrology, Tokyo Women’s Medical University, Tokyo, Japan; 9 Department of Pediatrics, National Hospital Organization Chibahigashi National Hospital, Chiba, Japan; 10 Department of Pediatric Nephrology, Japanese Red Cross Aichi Medical Center Nagoya Daini Hospital, Nagoya, Japan; 11 Faculty of Medicine, Department of Nephrology, Juntendo University, Tokyo, Japan; 12 Division of Nephrology, Department of Medicine, Showa University School of Medicine, Tokyo, Japan; 13 Department of Nephrology, Dokkyo Medical University Saitama Medical Center, Koshigaya, Japan; 14 Department of Metabolism, Endocrinology and Molecular Medicine, Osaka City University Graduate School of Medicine, Osaka, Japan; 15 Department of Nephrology and Rheumatology, Gunma University Graduate School of Medicine, Maebashi, Japan; 16 Health and Counseling Center, Osaka University, Toyonaka, Japan; 17 Department of Nephrology, Kanazawa Medical University School of Medicine, Uchinada, Japan; 18 Division of Clinical Nephrology and Rheumatology, Kidney Research Center, Niigata University Graduate School of Medical and Dental Sciences, Niigata, Japan; 19 Department of Nephrology, Osaka University Graduate School of Medicine, Suita, Japan; Shizuoka General Hospital: Shizuoka Kenritsu Sogo Byoin, JAPAN

## Abstract

The reclassification of membranoproliferative glomerulonephritis (MPGN) into immune-complex MPGN (IC-MPGN) and C3 glomerulopathy (C3G) based on immunofluorescence findings in kidney biopsies has provided insights into these two distinct diseases. C3G is further classified into dense deposit disease and C3 glomerulonephritis (C3GN) based on electron micrographic findings. Although these diseases have poor outcomes, limited Japanese literature confined to small, single-center cohorts exist on these diseases. We retrospectively analyzed 81 patients with MPGN type I and III from 15 hospitals in the Japan Renal Biopsy Registry to compare demographic, clinical characteristics and treatment outcomes of patients with IC-MPGN to those with C3GN. Of the 81 patients reviewed by immunofluorescence findings in kidney biopsies, 67 patients had IC-MPGN and 14 patients had C3GN. Age at diagnosis and systolic and diastolic pressure were higher and proteinuria and impaired renal function were significantly more prevalent in patients with IC-MPGN than those with C3GN. About 80% of the patients in both groups were treated with immunosuppressive therapy. At last follow-up (median 4.8 years), complete remission rate of proteinuria was significantly higher in patients with C3GN (64.3%) than in those with IC-MPGN (29.9%; P = 0.015). The renal survival rate was lower in patients with IC-MPGN when compared to C3GN (73.1% vs. 100%; log-rank, P = 0.031). Systolic blood pressure and renal function at baseline were independent predictors of progression to end-stage kidney disease. The overall prognosis of patients with C3GN is more favorable than for patients with IC-MPGN.

## Introduction

Membranoproliferative glomerulonephritis (MPGN) has previously been used as an umbrella term to describe a spectrum of hypocomplementemic glomerular diseases, which are a rare cause of end stage kidney disease (ESKD). More recently, MPGN has been reclassified into two diseases: immune-complex MPGN (IC-MPGN) and C3 glomerulopathy (C3G) based on immunofluorescence findings in kidney biopsies: predominant or exclusive C3 deposits in C3G and combined immunoglobulins and complement deposits in IC-MPGN [1]. C3G is further classified into dense deposit disease and C3 glomerulonephritis (C3GN) based on electron micrographic findings. However, these updated classification criteria do not specify how best to clinically differentiate these two diseases in terms of diagnosis, optimal treatment, and prognosis. Membranoproliferative glomerulonephritis describes a pattern of injury with characteristic mesangial cellularity and thickening of glomerular capillary walls due to subendothelial deposition of immune complexes or complement factors. Recent criteria to differentiate C3G from IC-MPGN is based on C3 predominance, rather than immunoglobulins, in tissues with an otherwise MPGN-like pattern of glomerular disease using immunofluorescence tests [2].

Beyond histologic differences, different molecular mechanisms underlie the two disease processes. These molecular processes may have diagnostic, therapeutic, and prognostic implications [3]. However, complement activation is central to the pathophysiology of both diseases [4]. The immune-complex MPGN possibly results from antigen-antibody immune complexes that activate the classical complement pathway where there is persistent antigenemia [2,5]. Adults with IC-MPGN commonly have antigens that result from infectious diseases, autoimmune diseases, or monoclonal gammopathies [5]. However, the cause of antigenemia in children with IC-MPGN is usually unknown [2]. In contrast, C3G is a dysregulation disorder of the primary alternative complement pathway [4]. In C3G, constitutive activation of the alternative complement cascade occurs due to impaired regulatory mechanisms that ultimately trigger downstream activation of the terminal complement cascade and membrane attack complex. C3G etiology is commonly attributed to autoantibodies’ formation that prevents degradation of C3 convertase (i.e., C3, C4, or C5 nephritic factors). Other causes of C3G include genetic mutations that lead to impaired functioning of alternative complement pathway regulators, at a low frequency in C3G (10%–25%), but some cases, which may reflect genetic mutations not yet known, are labeled as idiopathic [3,4].

Although both diseases have poor outcomes, it is difficult to directly compare the two entities due to the limited number of patients diagnosed with these conditions. Existing available literature has only been published in case series or is from small cohorts that are predominantly drawn from single-centers [6–8]. With more targeted therapeutic options on the horizon, particularly those that modulate the complement system, researchers ought to focus on further elucidating the clinical course of IC-MPGN and C3G in children.

We previously reported the clinicopathological findings of primary MPGN using the Japan Renal Biopsy Registry (J-RBR), a nationwide prospective registry system containing information from renal biopsies [9]. We illustrated that adult and elderly patients with primary MPGN had a higher prevalence of nephrotic syndrome, clinical hypertension, heavy proteinuria, and hypoalbuminemia at the time of biopsy when compared with children with primary MPGN [9]. However, our cross-sectional study could not establish a relationship between IC-MPGN and C3GN patients’ clinicopathological findings at diagnosis and their renal outcomes.

We therefore conducted a multicenter cohort study to describe the demographic, clinical, and laboratory characteristics and current initial treatment options for patients with IC-MPGN and C3GN in Japan using data from the J-RBR. We also described the predictive factors for renal function among IC-MPGN and C3GN patients, and the effects of different treatment modalities on renal function among these patients.

## Materials and methods

### Selection of patients from the J-RBR

**Fig 1:**
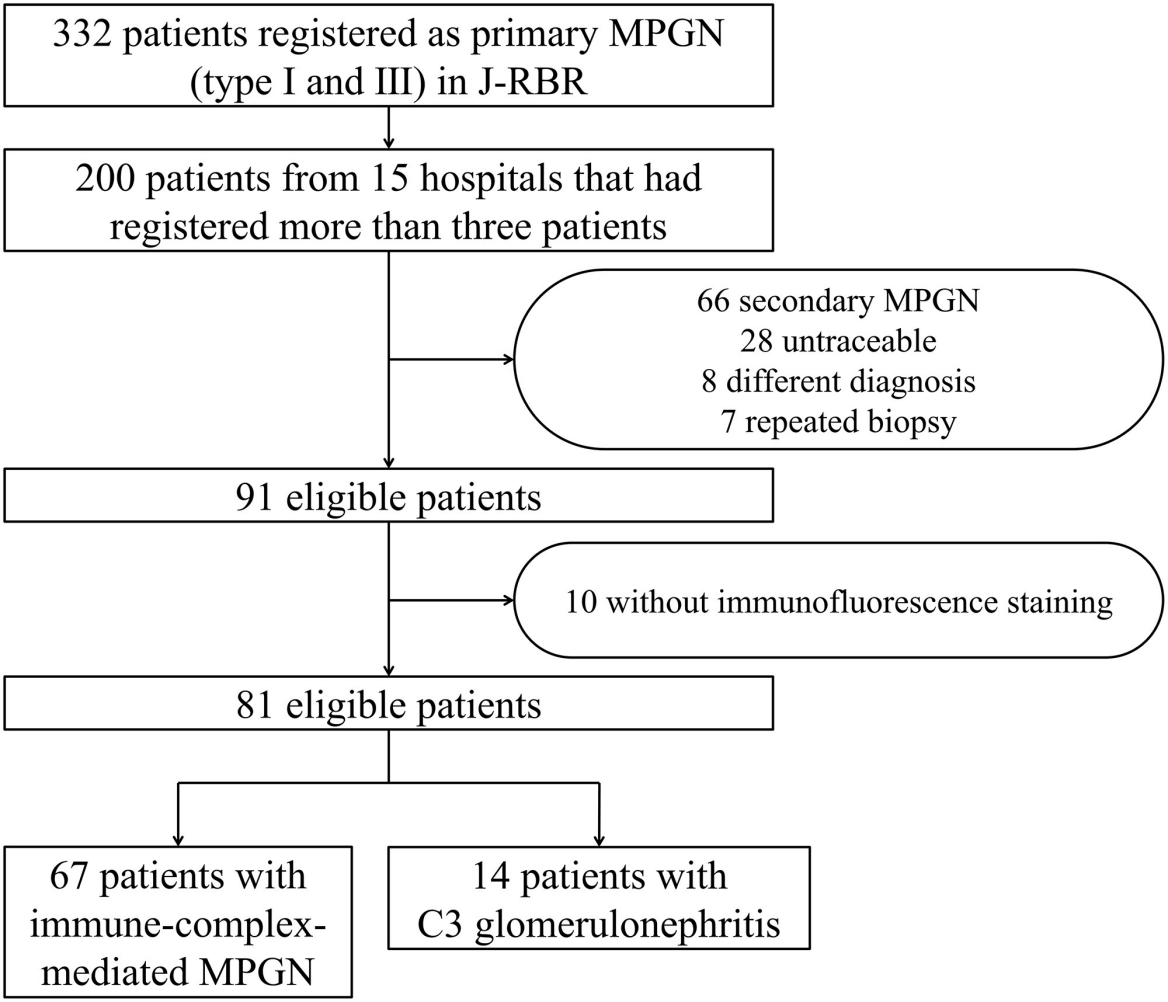
Flow chart showing enrollment of 81 patients. MPGN, membranoproliferative glomerulonephritis; J-RBR, Japan Renal Biopsy Registry.

The Committee for the Standardization of Renal Pathological Diagnosis and the Working Group for the Renal Biopsy Database of the Japanese Society of Nephrology established the J-RBR in 2007 [10]. Healthcare workers used the Internet Data and Information Center for Medical Research (INDICE) system of the University Hospital Medical Information System (UMIN) to register patient data on the J-RBR website. The J-RBR is registered under the Clinical Trial Registry of UMIN (Registration Number, UMIN000000618). The Ethics Review Board of the Japanese Society of Nephrology (JSN) approved the present study (JSN approval number 42; April 25, 2017) in accordance with the Declaration of Helsinki. Written informed consent for study participation was obtained from all the patients at the time of registration. Among the 26,535 patients with biopsy-proven disease registered in the J-RBR between July 2007 and June 2015, 593 (2.2%) patients were registered as having a histopathology of MPGN type I and III as we reported previously [9]. After excluding patients with “secondary” MPGN such as patients with lupus nephritis (n = 74), infectious disease (n = 72) including hepatitis B virus, hepatitis C virus and shunt infection, patients with immunoglobulin A (IgA) nephropathy (n = 29), and others (n = 86) [9], we selected 332 (1.3%) patients from 102 institutions with primary MPGN who had a histopathology of MPGN type I and III. From these, we identified 15 institutions that had registered more than 3 patients. Among 200 eligible patients from the 15 institutions, we excluded those with secondary MPGN (*N* = 66), those who had a different diagnosis possibly due to inputting error (*N* = 8), had more than one biopsy (*N* = 7), did not have immunofluorescence findings (*N* = 10), and were untraceable for more than 1 year (*N* = 28). We finally selected 81 patients who met our inclusion criteria (Fig 1), and categorized them to IC-MPGN and C3GN groups as per consensus guidelines from Kidney Disease: Improving Global Outcomes (KDIGO); C3G was defined as dominant C3 staining of at least two orders of magnitude higher than any other immune reactant in the glomeruli [11].

### Collection of clinical and pathological data from the J-RBR database and additional investigations

As described in detail in the previous study [10], the registered basic information, as well as urinary findings, blood findings, and blood pressure (BP), were assessed in the present study. Estimated glomerular filtration rates (eGFR) were calculated using the modified equation for Japanese adult [12] and children [13].

Clinical and pathological data were also collected from the J-RBR. Blood tests included complete blood counts, serum electrolytes (sodium, potassium, chloride, bicarbonate, phosphate, magnesium, and calcium), albumin, urea, creatinine, C3, C4, and CH50. Urine analyses included a urine dipstick test, protein, and creatinine. The presence of glomeruli with mesangial proliferative lesions, endocapillary proliferative lesions, and crescentic lesions and of interstitial fibrosis were collected from pathological reports as important pathological findings. Oral corticosteroids administered with their initial doses, the number of steroid pulse therapy courses, renin–angiotensin system (RAS) blockade, and immunosuppressive agents were documented as initial treatment modalities. Almost all urinary and blood items in J-RBR were re-examined during follow-up and/or at the final observation period. The presence or absence of end-stage kidney disease (ESKD) with renal replacement therapy (RRT), and all-cause death were used to evaluate patients’ prognoses.

### Evaluation of renal outcomes

The primary outcome was a decline in renal function as indicated by a 50% increase in serum creatinine (sCr) from baseline or ESKD with RRT with patients on hemodialysis, peritoneal dialysis, and renal transplant. The secondary outcome was the complete proteinuria remission rate, defined as a 24-h urinary protein of <0.3 g/day, or a urinary protein-to-creatinine ratio of <0.3 g/gCr [14,15], at the last known follow-up date. We also described the impact of steroid pulse therapy on decline in renal function or ESKD.

### Statistical analysis

Normally distributed continuous variables are expressed as mean ± standard deviation, and skewed continuous variables are expressed as median (interquartile range). Categorical variables are expressed as numbers (proportions). The two groups’ clinical parameters were compared using unpaired t-tests for normally distributed continuous variables, and Mann–Whitney U tests were used for skewed continued variables. Differences in proportions were evaluated using χ2 independent tests or Fisher exact tests. Renal outcomes were analyzed using the Kaplan–Meier method, and differences in survival curves were compared using log-rank tests. The Cox proportional hazards model was used to evaluate the impact of multiple covariates on a 50% increase in sCr and on progression to ESKD. Sex, treatment with steroid pulse therapy, and RAS blockade were included in the model as categorical variables, whereas age (per 10-year age bin), systolic blood pressure, amount of proteinuria, eGFR, and serum albumin were included in the model as continuous variables. The results of the multivariate analyses are expressed as hazard ratios, with 95% confidence intervals for a 50% increase sCr and for progression to ESKD. Variables with P < 0.1 on univariate analysis were included in the multivariable models. All data were analyzed using IBM SPSS v.26.0 (SPSS, Chicago, IL, USA), and p < 0.05 indicated a significant difference.

## Results

### Clinicopathological findings and initial treatment at the time of diagnosis

Of the 81 patients who had renal biopsy reports with immunofluorescence findings, 67 were classified as IC-MPGN, whereas the other 14 were classified as C3GN (Fig 1). Table 1 compares the demographic, clinical, laboratory findings at diagnosis and initial treatment modalities of patients with IC-MPGN to those with C3GN. The median age of patients with IC-MPGN and with C3GN was 62 and 19 years, respectively. Patients with IC-MPGN had a significantly higher systolic and diastolic blood pressure (140/76 vs. 113/65 mmHg), proteinuria values (3.89 vs. 0.77 g/day), worse renal function (sCr, 1.16 vs. 0.65 mg/dL; eGFR, 53.0 vs. 99.3 mL/min/1.73 m^2^), and lower serum albumin (2.9 vs. 3.7 g/dL) when compared to those with C3GN. The proportions of nephrotic syndrome (56.7 vs. 14.3%) were significantly higher, whereas those of chronic glomerular nephritis (38.8 vs. 85.7%) were significantly lower in patients with IC-MPGN when compared to patients with C3GN. When compared to IC-MPGN, patients with C3GN had significantly lower C3 (32.9 vs. 88.0 mg/dL), C4 (18.1 vs. 24.0 mg/dL), and CH50 (31.7 vs. 43.5 U/mL) at the time of diagnosis. The two patient groups did not differ by sex, body mass index, the degree of hematuria, and light microscopy findings.

**Table 1:** Clinicopathological findings at baseline and initial treatment between IC-MPGN and C3GN.

	IC-MPGN	C3GN	*P* value
***N***	67	14	
**Clinical characteristics at baseline**			
** Age (year)**	62 (29, 73)	19 (13, 26)	<0.001*
** Male, *N* (%)**	36 (53.7)	6 (42.9)	0.292
** Body mass index (kg/m** ^**2**^ **)**	22.5 ± 4.3	20.9 ± 4.0	0.175
** Systolic blood pressure (mmHg)**	140 ± 27	113 ± 11	0.001*
** Diastolic blood pressure (mmHg)**	76 ± 15	65 ± 9	0.006*
**First clinical manifestation, *N* (%)**			0.016*
** Nephrotic syndrome**	38 (56.7)	2 (14.3)	
** Rapidly progressive GN**	2 (3.0)	0 (0)	
** Acute GN**	1 (1.5)	0 (0)	
** Chronic GN**	26 (38.8)	12 (85.7)	
** Urinary protein (g/day) (or g/gCr)** ^a^	3.89 (1.14, 7.70)	0.77 (0.13, 4.10)	0.012*
** < 0.3 g/day (or g/gCr), *N* (%)**	5 (7.5)	5 (35.7)	0.021*
** 0.3–0.9**	10 (14.9)	3 (21.4)	
** 1.0–3.4**	18 (26.9)	2 (14.3)	
** ≥ 3.5**	34 (50.7)	4 (28.6)	
** Hematuria (/HPF)**			0.991
** < 5**	14 (20.9)	3 (21.4%)	
** 5–9**	12 (17.9)	2 (14.3%)	
** 10–29**	23 (34.3)	5 (35.7%)	
** > 30**	18 (26.9%)	4 (28.6%)	
** Serum creatinine (mg/dL)**	1.16 (0.70, 2.22)	0.65 (0.54, 0.91)	0.003*
** eGFR, mL/min/1.73 m** ^**2**^	53.0 ± 36.4	99.3 ± 36.6	<0.001*
** ≥ 90 mL/min/1.73 m** ^**2**^ **, *N* (%)**	11 (16.4)	8 (57.1)	0.005*
** 60–89**	13 (19.4)	5 (35.7)	
** 45–59**	7 (10.4)	0 (0.0)	
** 30–44**	13 (19.4)	1 (7.1)	
** 15–29**	17 (25.4)	0 (0.0)	
** < 15**	6 (9.0)	0 (0.0)	
** Total protein (g/dL)**	5.6 ± 0.8	6.1 ± 1.2	0.029*
** Serum albumin (g/dL)**	2.9 ± 0.8	3.7 ± 1.1	0.005*
** Serum total cholesterol (mg/dL)**	239 ± 80	229 ± 72	0.729
** Hemoglobin A1c (%)** ^b^	4.2 ± 2.2	4.7 ± 1.9	0.921
** Serum C3 (mg/dL)** ^b^	88.0 (70.0, 104.8)	32.9 (17.0, 72.9)	<0.001*
** Serum C4 (mg/dL)** ^b^	24.0 (17.1, 31.0)	18.1 (13.1, 21.0)	0.010*
** Serum CH50 (U/mL)** ^b^	43.5 (35.4, 52.5)	31.7 (12.0, 37.5)	0.013*
**Pathological findings, *N* (%)**			
** Mesangial proliferative GN**	43 (65.2)	10 (71.4)	0.652
** Endocapillary proliferative GN**	16 (24.2)	3 (21.4)	0.822
** Crescentic GN**	13 (19.7)	1 (7.1)	0.261
** Interstitial fibrosis**	32 (48.5)	4 (28.6)	0.174
**Initial treatment, *N* (%)**			
** Use of RAS blockers**	47 (70.1)	8 (57.1)	0.343
** Immunosuppressive therapy**	52 (77.6)	11 (78.6)	0.937
** Prednisolone**	50 (74.6)	11 (78.6)	0.756
** Initial dose (mg/kg/day)**	0.61 ± 0.23	0.68 ± 0.30	0.484
** Intravenous methylprednisolone**	29 (43.3)	7 (50.0)	0.484
** Cyclosporine**	17 (25.4)	2 (14.3)	0.373
** Cyclophosphamide**	7 (10.4)	0 (0.0)	0.206
** Mizoribine**	4 (6.0)	3 (21.4)	0.061
** Azathioprine**	2 (3.0)	1 (7.1)	0.454
** Mycophenolate mofetil**	3 (4.5)	0 (0.0)	0.420

Numbers are N (%) or mean ± standard deviation or median (25%, 75%).

IC-MPGN, immune-complex membranoproliferative glomerulonephritis; C3GN, C3 glomerulonephritis; eGFR, estimated glomerular filtration rate; HPF, high-power field; RAS, renin-angiotensin system.

*P < 0.05 for chi-square test, or Kruskal–Wallis test, as appropriate.

^a^Urinary protein/creatinine ratio (g/gCr) was used in 13 (19.4%) and 5 (35.7%) patients with IC-MPGN and C3GN, respectively, who had missing value of urinary protein (g/day).

^b^Number of missing value: HbA1c, *N* = 17 and 6 in IC-MPGN and C3GN; serum C3 and C4, *N* = 3 in IC-MPGN; serum CH50, *N* = 10 and 3 in IC-MPGN and C3GN.

An oral corticosteroid was the initial treatment for 75% of the patients in both groups, with an average initial dose of 0.61 mg/kg/day and 0.68 mg/kg/day for IC-MPGN and C3GN patients, respectively. From our analysis, 43.3% and 50.0% of patients with IC-MPGN and C3GN, respectively, received steroid pulse therapy; however, the number of administered courses ranged from one to three. Over 50% of patients in both groups received an RAS blockade. The initial therapies given to patients with IC-MPGN did not differ from that given to patients with C3GN.

### Renal outcomes

Table 2 compares the clinical findings, renal outcomes, and all-cause death at the last known follow-up of patients with IC-MPGN to those with C3GN. The median follow-up time was 4.6 and 6.3 years for patients with IC-MPGN and C3GN, respectively. At six months and last known follow-up date, the urinary protein of patients with IC-MPGN was significantly higher than that of patients with C3GN. At the last known follow-up date, patients with IC-MPGN had a lower complete remission rate (29.9 vs. 64.3%), worse renal function (sCr, 1.57 vs. 0.69 mg/dL and eGFR, 44.1 vs. 102.7 mL/min/1.73 m^2^), and lower serum albumin (3.5 vs. 4.4 g/dL) when compared to patients with C3GN.

**Table 2:** Clinical findings and outcomes after treatment between IC-MPGN and C3GN.

	IC-MPGN	C3GN	*P* value
***N***	67	14	
**Follow-up time (year)**	4.6 (1.8, 7.1)	6.3 (5.0, 9.0)	0.147
**6 months follow-up of proteinuria**	*N* = 54	*N* = 13	
** Urinary protein (g/day) (or g/gCr)**	0.74 (0.32, 2.61)	0.16 (0.04, 0.58)	0.011*
** < 0.3 g/day (or g/gCr), *N* (%)**	13 (24.1)	10 (76.9)	0.004*
** 0.3–0.9**	19 (35.2)	1 (7.7)	
** 1.0–3.4**	10 (18.5)	1 (7.7)	
** ≥ 3.5**	12 (22.2)	1 (7.7)	
**12 months follow-up of proteinuria**	*N* = 46	*N* = 13	
** Urinary protein (g/day) (or g/gCr)**	0.70 (0.11, 3.28)	0.16 (0.04, 1.16)	0.164
** < 0.3 g/day (or g/gCr), *N* (%)**	18 (39.1)	6 (53.8)	0.537
** 0.3–0.9**	7 (15.2)	3 (23.1)	
** 1.0–3.4**	13 (28.3)	2 (15.4)	
** ≥ 3.5**	8 (17.4)	1 (7.7)	
**Last known follow-up of proteinuria**	*N* = 51	*N* = 13	
** Urinary protein (g/day) (or g/gCr)**	0.70 (0.09, 3.74)	0.12 (0.06, 0.63)	0.025*
** < 0.3 g/day (or g/gCr), *N* (%)**	20 (39.2)	9 (69.2)	0.071
** 0.3–0.9**	7 (13.7)	3 (23.1)	
** 1.0–3.4**	11 (21.6)	1 (7.7)	
** ≥ 3.5**	13 (25.5)	0 (0.0)	
**Last Hematuria (/HPF)**	*N* = 51	*N* = 12	
** < 5**	37 (72.5)	9 (75.0)	0.307
** 5–9**	7 (13.7)	0 (0.0)	
** 10–29**	5 (9.8)	3 (25.0)	
** > 30**	2 (3.9)	0 (0.0)	
**Last serum creatinine (mg/dL)**	1.57 (0.70, 3.68)	0.69 (0.51, 0.92)	0.003*
**Last eGFR, mL/min/1.73 m** ^**2**^	44.1 ± 36.3	102.7 ± 40.1	<0.001*
**Last serum albumin (g/dL)**	3.5 ± 0.8	4.4 ± 0.4	<0.001*
**Final outcome at the last known follow-up date**			
** Complete remission**	17 (25.4)	9 (64.3)	0.015*
** Serum creatinine ×1.5**	29 (43.3)	0 (0.0)	0.002*
** End-stage kidney disease**	18 (26.9)	0 (0.0)	0.026*
** All-cause death** ^a^	12 (17.9)	0 (0.0)	0.078

^a^Outcomes: Malignancy four cases, pneumonia four cases, heart failure two cases, stroke one case, unknown one case. eGFR, estimated glomerular filtration rate; HPF, high-power field.

*P < 0.05 for chi-square test, Fisher’s exact test, ANOVA, or Kruskal–Wallis test, as appropriate.

**Fig 2:**
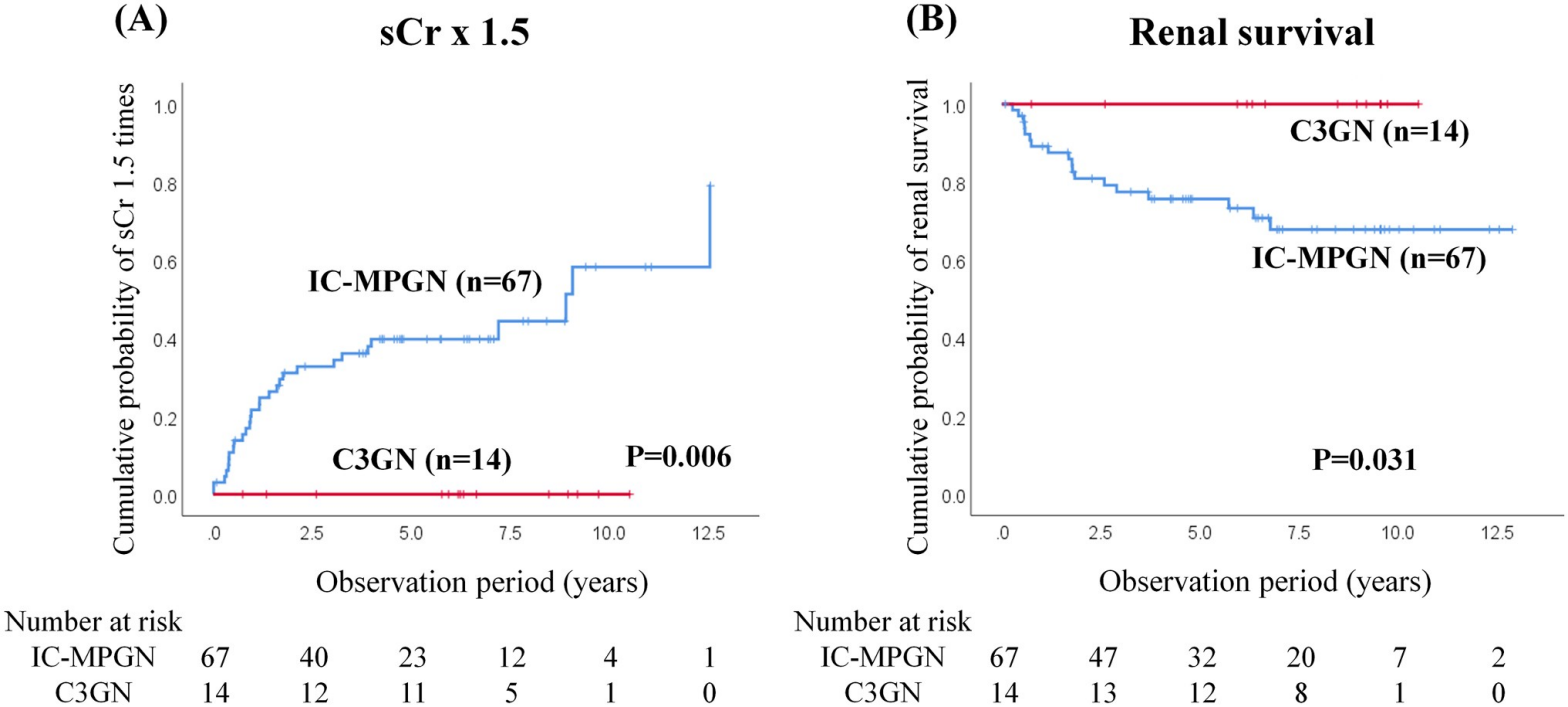
Renal outcomes of IC-MPGN and C3GN. Significant differences were observed in serum creatinine elevations of 50% (sCr × 1.5, A) and end-stage kidney disease (ESKD) (B) between IC-MPGN and C3GN patients. Kaplan-Meier curves for treated and untreated patients analyzed using log-rank tests. IC-MPGN, immune-complex membranoproliferative glomerulonephritis; C3GN, C3 glomerulonephritis.

During the observational period, no patients with C3GN attained a 50% increase in sCr, progressed to ESKD, or died. However, the rate of increase in sCr was significantly higher in patients with IC-MPGN than in those with C3GN (43.9% vs. 0.0% log-rank, P = 0.006) (Fig 2A). The renal survival rate significantly differed between patients with IC-MPGN and patients with C3GN (73.1% vs. 100%; log-rank, P = 0.031) (Fig 2B). Although 12 (17.9%) patients with IC-MPGN died (4 patients with malignancy, 4 patients with pneumonia, 2 patients with heart failure, 1 patient with stroke, and 1 patient with an unknown diagnosis), there was significant difference in the all-cause mortality between patients with IC-MPGN and those with C3GN.

**Fig 3:**
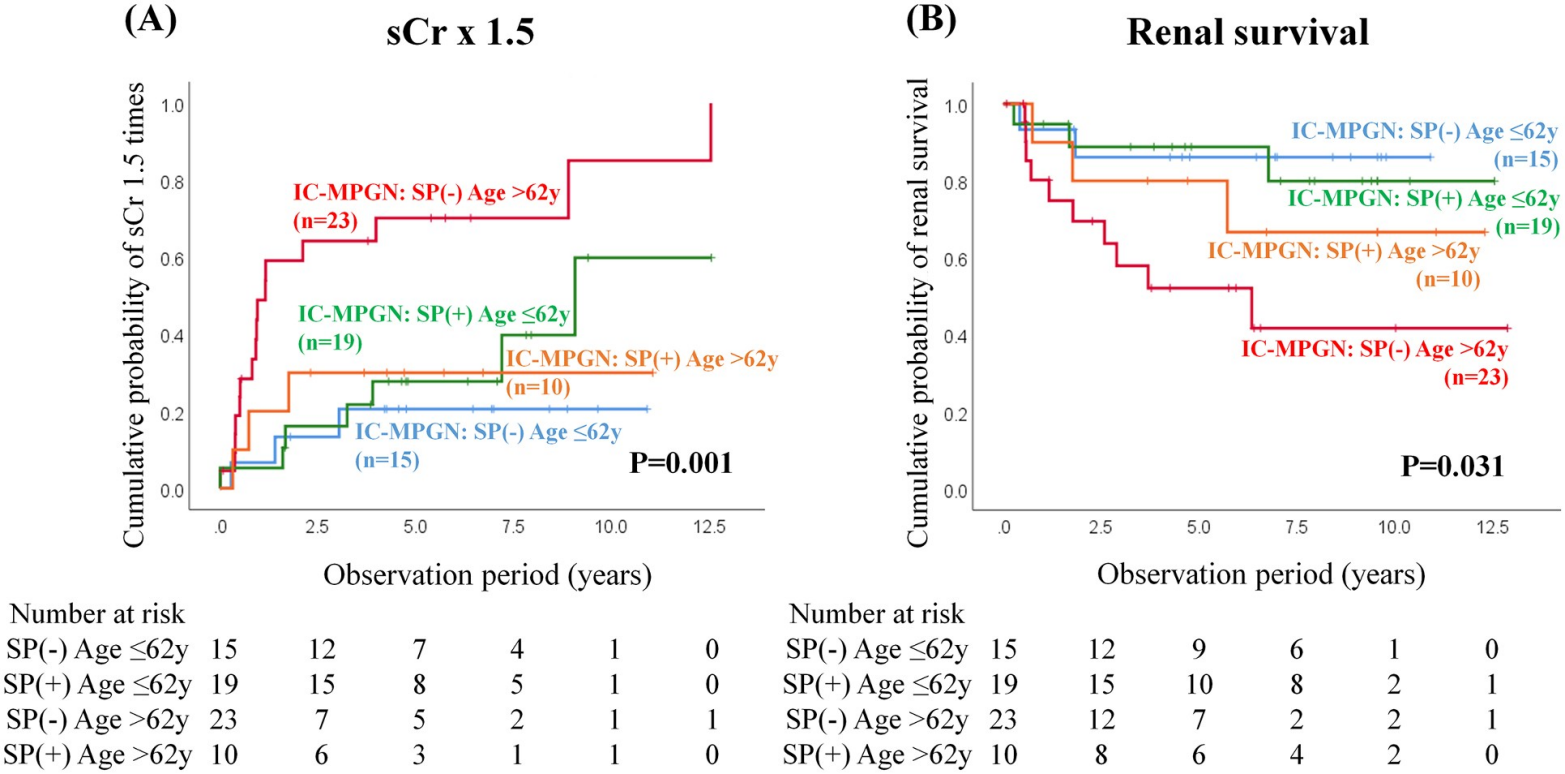
Renal outcomes of IC-MPGN patients with or without steroid pulse therapy stratified by median age (62 y) at onset in serum creatinine elevations of 50% (sCr × 1.5, A) and end-stage kidney disease (B). Kaplan-Meier curves for treated and untreated patients analyzed using log-rank tests. IC-MPGN, immune-complex membranoproliferative glomerulonephritis; SP, steroid pulse.

We then examined the impact of steroid pulse therapy stratified by median age (62 y) on decline in renal function (sCr x1.5) or ESKD in IC-MPGN using Kaplan–Meier curves (Fig 3). The subgroup of older age without steroid pulse therapy had significantly poorer outcomes, i.e., both a decline in renal function (Fig 3A, log-rank P = 0.001) and ESKD (Fig 3B, log-rank P = 0.031).

Table 3 shows the IC-MPGN patient’ clinicopathological findings at baseline based on whether steroid pulse therapy was administered at the time of initial treatment stratified by median age (62 y). The younger patients with IC-MPGN showed lower systolic blood pressure levels, and urinary proteinuria, higher eGFR levels, and serum albumin levels than the older patients with IC-MPGN.

**Table 3:** Clinicopathological findings at baseline in patients with IC-MPGN stratified by median age (62 y) and with or without steroid pulse therapy at initial treatment.

	≤ 62y		> 62y		*P* value
	Steroid pulse (-)	Steroid pulse (+)	Steroid pulse (-)	Steroid pulse (+)	
***N***	15	19	23	10	
**Age (year)**	43 (17, 54)	23 (12, 40)	76 (68, 81)	69 (66, 76)	<0.001*
**Male, *N* (%)**	7 (46.7)	11 (57.9)	11 (47.8)	7 (70.0)	0.610
**Body mass index (kg/m** ^**2**^ **)**	21.9 ± 3.2	20.7 ± 4.9	23.5 ± 4.7	24.6 ± 2.2	0.078
**Systolic BP (mmHg)**	132.8 ± 29.3	123.3 ± 27.3	152.8 ± 20.6	155.9 ± 14.8 ^#^	0.001*
**Diastolic BP (mmHg)**	79.3 ± 19.9	71.2 ± 16.0	78.1 ± 11.7	77.4 ± 10.2	0.215
**Urinary protein (g/day) (or g/gCr)**	2.03 (0.40, 3.90)	4.53 (1.14, 6.49)	3.89 (0.79, 8.96)	7.21 (4.30, 11.1)	0.041*
**Serum creatinine (mg/dL)**	0.88 (0.64, 2.40)	0.75 (0.46, 2.10)	1.50 (1.05, 1.93)	1.89 (1.20, 3.96)	0.028*
**eGFR (mL/min/1.73 m** ^**2**^ **)**	65.8 ± 42.5	74.2 ± 40.5	37.2 ± 18.8	29.6 ± 17.8 ^#^	0.004*
**Total protein (g/dL)**	6.1 ± 0.6	5.5 ± 1.1	5.5 ± 0.8	5.4 ± 0.6	0.047*
**Serum albumin (g/dL)**	3.4 ± 0.5	2.9 ± 1.1	2.6 ± 2.4 ^¶^	2.7 ± 0.3	0.005*
**Serum total cholesterol (mg/dL)**	220.8 ± 76.2	256.8 ± 92.9	241.4 ± 66.1	233.9 ± 96.1	0.495
**Hemoglobin A1c (%)**	4.5 ± 1.3	4.9 ± 1.3	4.1 ± 2.2	4.8 ± 2.7	0.263
**Serum C3 (mg/dL)**	77.5 (50.3, 103.3)	84.0 (44.0, 91.0)	96.5 (89.5, 114.0)	77.0 (56.0, 96.0)	0.013*
**Serum C4 (mg/dL)**	25.0 (14.5, 32.9)	16.6 (13.0, 24.8)	27.2 (22.9, 33.3)	21.5 (19.3, 32.0)	0.019*
**Serum CH50 (U/mL)**	26.1 (23.3, 51.2)	39.2 (31.3, 44.2)	50.3 (41.4, 56.8)	43.1 (33.6, 57.3)	0.007*
**Mesangial proliferative GN**	7 (46.7)	15 (78.9)	16 (69.6)	5 (50.0)	0.107
**Endocapillary proliferative GN**	2 (13.3)	6 (31.6)	4 (17.4)	4 (40.0)	0.293
**Crescentic GN**	0 (0.0)	4 (21.1)	6 (26.1)	3 (30.0)	0.171
**Interstitial fibrosis**	6 (40.0)	7 (36.8)	14 (60.9)	5 (50.0)	0.472
**Use of RAS blockers**	9 (60.0)	13 (68.4)	17 (73.9)	8 (80.0)	0.709

Numbers are N (%) or mean ± standard deviation or median (25%, 75%). BP, blood pressure; eGFR, estimated glomerular filtration rate; GN, glomerulonephritis; RAS, renin-angiotensin system.

*P < 0.05 for chi-square test, or Kruskal–Wallis test, as appropriate (followed by multiple comparison test with the Bonferroni correction).

^¶^*P* < 0.05 for comparison of younger patients without steroid pulse therapy;

^#^*P* < 0.05 for comparison of younger patients with steroid pulse therapy.

Table 4 shows the IC-MPGN patient’ outcomes at the last known follow-up based on whether steroid pulse therapy was administered at the time of initial treatment stratified by median age (62 y).Although the subgroup of older patients with steroid pulse therapy was highest urinary proteinuria and lowest eGFR at baseline (Table 3), the both outcome of a 50% increase in creatinine and ESKD was better than the subgroup of older patients without steroid pulse therapy (Table 4), suggesting that some patients with IC-MPGN, even though older patients, might be amenable to treatment with steroid pulse therapy. Additionally, the ratio with steroid pulse therapy in the subgroup of older patients appeared lower than that of the subgroup of younger patients (Table 4). One possible reason is that the baseline age was highest in the subgroup of older patients without steroid pulse therapy, therefore this therapy might be avoided in those patients, because treatment with glucocorticoids and nephrotic syndrome itself often make patients susceptible to infection [14].

**Table 4:** Outcomes in patients with IC-MPGN stratified by median age (62 y) and with or without steroid pulse therapy at initial treatment.

	≤ 62y		> 62y		*P* value
	Steroid pulse (-)	Steroid pulse (+)	Steroid pulse (-)	Steroid pulse (+)	
***N***	15	19	23	10	
**Last urinary protein (g/day)(or g/gCr)**	0.53 (0.08, 0.92)	0.09 (0.05, 0.43)	3.15 (1.15, 4.57)	2.91 (0.26, 4.55)	0.001*
**Last serum creatinine (mg/dL)**	0.89 (0.68, 1.79)	0.87 (0.53, 2.28)	3.37 (1.33, 5.03)	1.81 (0.84, 4.85)	0.005*
**Last eGFR (mL/min/1.73 m** ^**2**^ **)**	58.7 ± 35.1	67.2 ± 39.9	21.9 ± 20.0 ^¶^	34.9 ± 30.2	0.001*
**Last serum albumin (g/dL)**	3.9 ± 0.8	3.8 ± 0.8	3.1 ± 0.6 ^¶^	3.1 ± 0.8	0.004*
**Complete remission**	2 (13.3)	9 (47.4)	2 (8.7)	2 (20.0)	0.038*
**Serum creatinine ×1.5**	2 (13.3)	7 (36.8)	17 (73.9)	3 (33.3)	0.002*
**End-stage kidney disease**	2 (13.3)	3 (15.8)	10 (43.5)	3 (33.3)	0.092
**All-cause death**	1 (6.7)	1 (5.3)	7 (30.4)	3 (13.3)	0.065

Numbers are N (%) or mean ± standard deviation or median (25%, 75%). eGFR, estimated glomerular filtration rate.

*P < 0.05 for chi-square test, or Kruskal–Wallis test, as appropriate (followed by multiple comparison test with the Bonferroni correction).

^¶^*P* < 0.05 for comparison of younger patients without steroid pulse therapy.

Clinicopathological findings at baseline and outcomes in patients with C3GN stratified by median age (19 y) are shown in S1 and S2 Tables, respectively. Although the ratio of interstitial fibrosis at baseline renal biopsy was significantly higher in the older subgroup than the younger subgroup, there were no significant differences for other clinicopathological findings at baseline and outcomes between the two subgroups (S1 and S2 Tables).

### Factors affecting a decline in renal function

Finally, we examined the factors affecting a decline in renal function in IC-MPGN. Univariate analyses identified increasing age (per 10 years), higher systolic blood pressure, lower eGFR, and lower serum albumin values as prognostic factors for a decline in renal function. Subsequently, multivariate analyses retained lower eGFR values as prognostic factors for a decline in renal function (Table 5). Similarly, older patients (per 10 years), higher systolic blood pressure, and lower eGFR were independent predictive factors for progression to ESKD in the univariate analysis, whereas higher systolic blood pressure and lower eGFR were also significant independent prognostic factors for the progression to ESKD in the multivariate analyses (Table 5).

**Table 5:** Association of Serum creatinine×1.5 and progression to end-stage kidney disease in patients with IC-MPGN.

	Serum creatinine×1.5 after therapy						Progression to end-stage kidney disease					
	Univariate			Multivariate			Univariate			Multivariate		
	HR	95%CI	*P*	HR	95%CI	*P*	HR	95%CI	*P*	HR	95%CI	*P*
**Age (per 10 years)**	1.306	(1.087–1.569)	0.004*	1.009	(0.801–1.271)	0.937	1.437	(1.088–1.899)	0.011*	1.073	(0.765–1.505)	0.683
**Gender (Male vs. Female)**	0.750	(0.345–1.634)	0.469	–	–	–	0.944	(0.365–2.439)	0.905	–	–	–
**Systolic BP (mmHg)**	1.026	(1.011–1.042)	0.001*	1.009	(0.988–1.030)	0.417	1.039	(1.018–1.061)	<0.001*	1.033	(1.006–1.060)	0.015*
**Urinary protein (g/day or g/gCr)**	1.059	(0.987–1.135)	0.111	–	–	–	1.078	(0.989–1.174)	0.088	1.008	(0.910–1.116)	0.883
**eGFR (ml/min/1.73m** ^**2**^ **)**	0.970	(0.954–0.986)	<0.001*	0.966	(0.945–0.988)	0.002*	0.933	(0.899–0.969)	<0.001*	0.928	(0.888–0.970)	0.001*
**Serum albumin (g/dL)**	0.589	(0.366–0.947)	0.029*	0.523	(0.258–1.059)	0.072	0.727	(0.400–1.321)	0.296	–	–	–
**Steroid pulse therapy (ref. no use)**	0.528	(0.245–1.138)	0.103	–	–	–	0.545	(0.204–1.455)	0.225	–	–	–
**RAS inhibitors (ref. no use)**	1.288	(0.567–2.929)	0.545	–	–	–	2.313	(0.669–7.966)	0.185	–	–	–

*Statistically significant. BP, blood pressure; eGFR, estimated glomerular filtration rate; RAS, renin-angiotensin system.

## Discussion

This study presents the clinical and laboratory characteristics and clinical outcomes of patients form the largest Japanese IC-MPGN/C3GN cohort to date drawn from a multicenter, nationwide study based on the J-RBR. Although patients with C3GN had mild clinical and laboratory findings at baseline, they had a more favorable prognosis following treatment when compared to those with IC-MPGN. On the contrary, IC-MPGN patients treated with steroid pulse therapy at initial diagnosis had a significantly higher complete proteinuria remission rate than IC-MPGN patients who did not receive steroid pulse therapy. A patient’s baseline systolic blood pressure and renal function were independent predictors of progression to ESKD, whereas a patient’s baseline renal function and serum albumin were independent predictors of declining renal function (50% increase in sCr from baseline).

The clinical features of the patients in this study illustrated that nephrotic syndrome was more frequent in IC-MPGN (56.7%) than in C3GN (14.3%) patients. These findings concur with previously reported pediatric and adult studies that showed that nephrotic syndrome is more frequent in patients with IC-MPGN (43%–70%) when compared to patients with C3GN (26%–52%) [3,16]. We also found that proteinuria was more prevalent in patients with IC-MPGN than in patients with C3GN; similar findings were seen with hypoalbuminemia despite there being no differences in pathological findings (in terms of extent of mesangial and endocapillary hypercellularity, crescent formations, and interstitial fibrosis) between the two patient groups.

Controversy exists about whether an IC-MPGN and C3GN patient’s clinical findings at diagnosis can predict his or her long-term renal prognosis. This conflict is due to heterogeneous MPGN definitions in studies published before and shortly after the reclassification of these disease entities. Furthermore, studies performed after disease reclassification were confined to pediatric populations and had small sample sizes. The majority of data on IC-MPGN outcomes has been drawn from adult literature. These data show that the disease invariably results in renal deterioration with most patients reaching ESKD within a decade of diagnosis. Moreover, there has been no improvement in patient outcomes over the last several decades [17,18]. The multiple small cohort pediatric research studies in children with IC-MPGN/C3G, conducted post reclassification, have demonstrated either a worse renal prognosis in C3G [6,7], or no differences in treatment outcomes between the groups [8,19–21]. Bomback et al. previously found a 40% rate of progression rate to advanced chronic kidney disease (CKD), ESKD, or death in a large American cohort study of 111 C3G patients; approximately one-third of the 111 patients were children [22]. Iatropoulos et al. reported that nephrotic syndrome and severe histological damage diagnosed at disease onset had the possibility of increasing the risk of progression to ESKD in a large Italian cohort of 140 adult and pediatric patients with idiopathic Ig-MPGN and C3G [16]. In our study, higher systolic blood pressure and lower eGFR at baseline were significant independent prognostic factors for progression to ESKD, suggesting that blood pressure and renal function at baseline were strong predictors for renal outcome in IC-MPGN. These results are consistent with data in children that showed that hypertension and renal dysfunction at initial presentation are independent risks factor for poor renal outcomes [20].

In our study, serum C3, C4, and CH50 were significantly lower in patients with C3G than in patients with IC-MPGN. This finding suggests that both the alternative complement pathway and classical complement pathway are more activated in patients with C3G than in those with IC-MPGN. Previous studies show that low serum C4 level, which is a marker of classical pathway activation, is rarely (<5%) found in patients with IC-MPGN, which is in sharp contrast to secondary forms of IC-MPGN [16,23]. This finding suggests that secondary MPGN might be excluded in our cohort.

IC-MPGN management is still largely untargeted and centers on treatment of the underlying cause, alongside RAS blockade in proteinuric patients and the use of corticosteroids and either oral cyclophosphamide or mycophenolate mofetil to treat severe progressive IC-MPGN [3,11]. In our cohort, there were no significant differences in the administration of steroids or immunosuppressive agents between IC-MPGN and C3GN. However, when we focused on the presence or absence of steroid pulse therapy, we found that IC-MPGN patients treated with steroid pulse therapy at initial diagnosis were younger, had lower C3 and C4 levels and subsequently higher complete proteinuria remission rate than those who did not receive steroid pulse therapy. As previously mentioned, some patients with primary complement dysregulation might be amenable to treatment even in patients with IC-MPGN. Terminal complement blockade using eculizumab, which is an anti-C5 monoclonal antibody, has been attempted in patients with C3G with mixed outcomes [24–26]; targeted therapy for this disease, therefore remains an area of interest.

Regarding the histologic reclassification, nearly one-third of the patients originally diagnosed as primary MPGN were reclassified as secondary MPGN. These patients included those with underlying infectious diseases, autoimmune diseases, or paraproteins which were difficult to diagnose at the time of registration. To address the J-RBR registration system’s limitations, the revised version 2018 of the J-RBR/J-KDR system attempts to include all kidney diseases by focusing on their pathogenesis [27]. The new platform will help standardize the registration of cases of kidney biopsy to systematically collect higher quality data. Based on these advances, it is more desirable to have a classification based on etiology and pathogenesis.

Although a clinical trial is preferable to address the lack of evidence in this field, it is difficult to study the outcomes of rare renal disorders such as IC-MPGN and C3G. In fact, primary MPGN accounts for only 2.6% of all renal biopsies listed in the J-RBR between 2009 and 2010 [28]. The present multicenter study was made feasible by the J-RBR’s accessible large database. The J-RBR was the first nationwide, prospective registry of renal biopsies that lists 5,000 patients from 130 institutions in Japan annually since it was established in 2007 [10]. The registry contributes to the standardization of histological diagnoses and classification, and facilitates nationwide epidemiological studies of renal pathologies, such as nephrotic syndrome and glomerulonephritis. Moreover, there are plans to conduct a secondary cohort study on nephrotic syndrome in elderly patients, similar to this study, using the J-RBR database [29,30]. and on patients with IgA vasculitis with nephritis [31]. Secondary applications of the J-RBR database will increasingly become important in the future.

The strength of this study was that it evaluated a large Japanese IC-MPGN/C3G cohort of patients in a multicenter, nationwide study based on data from a renal biopsy registry. Therefore, this study’s results are representative of the current situation in Japan. However, this study has some limitations. Firstly, it employed a retrospective design with a short observation period of 4.8 years since the database was established in 2007. Although we tried to control for confounding factors using multivariate analysis, our sample size was suboptimal, and we did not consider all potential comorbidities. Generally, the number of patients affected with rare diseases is so limited that it is difficult to collect their specimens and information. Therefore, we plan to create as large a cohort as possible of patients with IC-MPGN and C3GN and follow them prospectively according to a fixed protocol. Secondly, this registry did not contain detailed information on complement assays (i.e., soluble C5b9, C3d, and factors H and I) and genetic factors. However, only 20% of patients with C3G are likely to have a known genetic mutation [3,16]. Therefore, it is unlikely that the absence of this information would have an impact on our findings.

In summary, Japanese patients with C3GN have a more favorable clinical course compared to patients with IC-MPGN. Several newly developed anti-complement agents are now available. Therefore, anti-complement therapies present a realistic therapeutic option for complement-related diseases. Future studies in children and adults with IC-MPGN and C3GN should investigate sequential measurements of serum complement activity and autoantibody titres as well as genetic analysis as prognostic biomarkers of disease in a prospective longitudinal cohort study. Such studies should also further delineate the pathophysiology and possible treatment outcomes after targeted molecular therapies.

## Supporting information

S1 Table Clinicopathological findings at baseline in patients with C3GN stratified by median age (19 y). (DOCX)

S2 Table Outcomes in patients with C3GN stratified by median age (19 y). (DOCX)
