# Supplementary material for: Demographic, clinical characteristics and treatment outcomes of immune-complex membranoproliferative glomerulonephritis and C3 glomerulonephritis in Japan: A retrospective analysis of data from the Japan Renal Biopsy Registry
Source: PLoS One. 2021 Sep 14;16(9):e0257397. doi: 10.1371/journal.pone.0257397 (PMC8439563; doi:10.1371/journal.pone.0257397)
Supplement: S1 Table — (DOCX) [file pone.0257397.s001.docx]

**S1 Table. Clinicopathological findings at baseline in patients with C3GN stratified by median age (19 y).**

|  | **≤ 19y** | **> 19y** | ***P* value** |
| --- | --- | --- | --- |
| ***N*** | 7 | 7 |  |
| **Age (year)** | 13 (10, 16) | 26 (22, 54) | 0.001^*^ |
| **Male, *N*(%)** | 5 (71.4) | 3 (42.9) | 0.296 |
| **Body mass index (kg/m^2^)** | 20.5 ± 4.6 | 21.3 ± 3.6 | 0.949 |
| **Systolic BP (mmHg)** | 113.9 ± 12.9 | 112.3 ± 9.2 | 0.535 |
| **Diastolic BP (mmHg)** | 63.4 ± 10.9 | 67.4 ± 5.9 | 0.259 |
| **Urinary protein (g/day) (or g/gCr)** | 0.80 (0.30, 1.20) | 1.60 (0.49, 4.54) | 0.165 |
| **Serum creatinine (mg/dL)** | 0.55 (0.38, 0.84) | 0.90 (0.64, 1.29) | 0.128 |
| **eGFR (mL/min/1.73 m^2^)** | 107.1 ± 29.1 | 91.5 ± 43.6 | 0.318 |
| **Total protein (g/dL)** | 6.1 ± 1.3 | 6.1 ± 1.2 | 0.902 |
| **Serum albumin (g/dL)** | 3.7 ± 1.2 | 3.6 ± 1.1 | 0.902 |
| **Serum total cholesterol (mg/dL)** | 211.3 ± 97.6 | 246.1 ± 29.6 | 0.128 |
| **Hemoglobin A1c (%)** | 5.1 ± 0.4 | 4.5 ± 2.2 | 0.733 |
| **Serum C3 (mg/dL)** | 35.8 (13.8, 45.0) | 44.0 (29.5, 75.9) | 0.902 |
| **Serum C4 (mg/dL)** | 11.3 (9.3, 18.9) | 20.1 (17.9, 21.3) | 0.053 |
| **Serum CH50 (U/mL)** | 33.1 (10.0, 42.0) | 30.7 (21.2, 43.5) | 0.537 |
| **Mesangial proliferative GN** | 6 (85.7) | 4 (57.1) | 0.280 |
| **Endocapillary proliferative GN** | 2 (28.6) | 1 (14.3) | 0.500 |
| **Crescentic GN** | 1 (14.3) | 0 (0.0) | 0.500 |
| **Interstitial fibrosis** | 0 (0.0) | 4 (57.1) | 0.035^*^ |
| **Use of RAS blockers** | 2 (28.6) | 6 (85.7) | 0.051 |

Numbers are N (%) or mean ± standard deviation or median (25%, 75%). BP, blood pressure; eGFR, estimated glomerular filtration rate; GN, glomerulonephritis; RAS, renin-angiotensin system.

^*^*P* < 0.05 for chi-square test, or Kruskal–Wallis test, as appropriate.
