# Supplementary material for: Demographic, clinical characteristics and treatment outcomes of immune-complex membranoproliferative glomerulonephritis and C3 glomerulonephritis in Japan: A retrospective analysis of data from the Japan Renal Biopsy Registry
Source: PLoS One. 2021 Sep 14;16(9):e0257397. doi: 10.1371/journal.pone.0257397 (PMC8439563; doi:10.1371/journal.pone.0257397)
Supplement: S2 Table — (DOCX) [file pone.0257397.s002.docx]

**S2 Table. Outcomes in patients with C3GN stratified by median age (19 y).**

|  | **≤ 19y** | **> 19y** | ***P* value** |
| --- | --- | --- | --- |
| ***N*** | 7 | 7 |  |
| **Last urinary protein (g/day)(or g/gCr)** | 0.24 ± 0.29 | 0.51 ± 0.76 | 0.445 |
| **Last serum creatinine (mg/dL)** | 0.59 (0.51, 0.65) | 0.94 (0.49, 1.07) | 0.329 |
| **Last eGFR (mL/min/1.73 m^2^)** | 109.8 ± 21.7 | 94.1 ± 57.1 | 0.329 |
| **Last serum albumin (g/dL)** | 4.6 ± 0.4 | 4.1 ± 0.4 | 0.082 |
| **Complete remission** | 5 (71.4) | 4 (57.1) | 0.591 |
| **Serum creatinine ×1.5** | 0 (0.0) | 0 (0.0) | ― |
| **End-stage kidney disease** | 0 (0.0) | 0 (0.0) | ― |
| **All-cause death** | 0 (0.0) | 0 (0.0) | ― |

Numbers are N (%) or mean ± standard deviation or median (25%, 75%). eGFR, estimated glomerular filtration rate.
